# Protocrystallinity of Monodispersed Ultra-Small Templated Mesoporous Silica Nanoparticles

**DOI:** 10.3390/nano14121052

**Published:** 2024-06-19

**Authors:** Laurent Bonneviot, Belén Albela, Feifei Gao, Pascal Perriat, Thierry Epicier, Mohamad El Eter

**Affiliations:** 1 Laboratoire de Chimie, Ecole Normale Supérieure de Lyon, Université de Lyon, 69364 Lyon, France; 2 Matériaux—Ingénierie et Sciences (MATEIS) Unité Mixte de Recherche CNRS, INSA de Lyon, 69621 Villeurbanne, Francethierry.epicier@univ-lyon1.fr (T.E.); 3 College of Arts and Sciences, American University of Irak, Bagdad, Iraq

**Keywords:** mesoporous silica, nanoparticles, crystallinity, synthesis, monodispersion, porosity

## Abstract

Monodisperse and semi-faceted ultra-small templated mesoporous silica nanoparticles (US-MSNs) of 20–25 nm were synthesized using short-time hydrolysis of tetraethoxysilane (TEOS) at room temperature, followed by a dilution for nucleation quenching. According to dynamic light scattering (DLS), a two-step pH adjustment was necessary for growth termination and colloidal stabilization. The pore size was controlled by cetyltrimethylammonium bromide (CTAB), and a tiny amount of neutral surfactant F127 was added to minimize the coalescence between US-MSNs and to favor the transition towards internal ordering. Flocculation eventually occurred, allowing us to harvest a powder by centrifugation (~60% silica yield after one month). Scanning transmission electron microscopy (STEM) and 3D high-resolution transmission electron microscopy (3D HR-TEM) images revealed that the US-MSNs are partially ordered. The 2D FT transform images provide evidence for the coexistence of four-, five-, and sixfold patterns characterizing an “on-the-edge” crystallization step between amorphous raspberry and hexagonal pore array morphologies, typical of a protocrystalline state. Calcination preserved this state and yielded a powder characterized by packing, developing a hierarchical porosity centered at 3.9 ± 0.2 (internal pores) and 68 ± 7 nm (packing voids) of high potential for support for separation and catalysis.

## 1. Introduction

There is a lot of research on the size control of porous particles in the nanometric scale range below 100 nm. As stable colloidal suspensions, they provide an alternative to liquid crystalline lipid or polymeric nanocarriers for magnetic or optical labeling and vectorization, which are useful for imaging and drug delivery in medical diagnosis and therapy [1,2,3,4,5]. These nanosized objects can also be applied as building units to create hierarchically structured porosity to minimize internal molecular diffusion for the design of adsorbents and catalysts. This is useful in many different fields such as the industrial production of chemicals and pollution control. In both cases, well-defined physical properties, such as monodispersion and size calibration, are key points for better diffusion control in living systems and for optimal pharmacological use. Sizes smaller than 100 nm are required to avoid capillary plugging in living systems (embolism). Most of the inorganic nanocargos that have been developed using inorganic matrices are mainly designed from colloidal suspensions of non-porous gold nanoparticles functionalized on their external surface; they are already in the ultra-small size range, i.e., below 20 nm [6,7]. Silica-based nanocargos have also been developed due to their well-defined internal porosity in the micropore (<2 nm for zeolite) or mesopore (2 to 50 nm for micelle-templated material) ranges. Despite being of interest for their molecular retention and controlled diffusivity, there are very scarce examples of combining fine tunings of both porosity and grain size at such a fine scale of length [1,8,9]. The first article on mesoporous nanoparticles was published in 2001, reporting polydispersed mesostructured silicas (20 to 100 nm), with a regular array of channels observed only for particles larger than 50–60 nm [10]. The synthesis was inspired by a recipe of bulk periodic mesoporous silica (PMS) of MCM-41 or MCM-48 types using a cationic surfactant cetyltrimethylammonium as a pore structuring agent [10,11,12]. Up to now, mesoporous silica nanoparticles with well-defined cubic or hexagonal mesophases have been reported for sizes larger than 70 nm [13]. These systems exhibit attractive properties, such as high pore volume, large specific surface area, and surfaces that are easy to functionalize from their silanol groups [14,15]. To keep the size as small as possible, the first parameter to take into account is supersaturation, which determines the number of nuclei from which growth takes place. Accordingly, nucleation quenching using dilution was the first approach adopted [10,11,12]. Chemical quenching using functionalized organosilanes was also applied since it blocks the polycondensation and, depending on the organic function (thiol or amine), also controls the surface charges, stabilizing small sizes and yielding different morphologies [11,16,17,18,19]. Furthermore, the stabilization of nanoparticles in the range of 25–200 nm using ammonia, ethanol, and triethanolamine (TEA) additives has also been reported [16,20,21]. In particular, TEA additives have been claimed to act both as a base and a chelating agent for the oligomeric silica precursors, yielding nanosized particles (20–40 nm) that spontaneously merge one with another into hierarchical porous silica materials [16]. Using the same additive, Bein et al. successfully stabilized colloidal solutions of well-controlled size and shape nanoparticles in high yield and, notably without the need for dilution [21]. However, these spherical nanoparticles exhibit little ordering and weak X-ray diffraction (XRD) diffraction [16,22,23]. This is due to a radial array of channels, which appears suitable for drug delivery since it allows size control even in the presence of organosilanes [16,17,18,19,24]. The resistance of such radially structured mesoporous nanoparticles towards dilution in water or physiological media remains limited to a few days, which may present an advantage in some medical applications like diagnosis by imaging or short-time drug delivery control [25]. Fine pore structure tuning from radial arrays to raspberry and worm-like shapes has been obtained using polyolamine at the appropriate concentrations, although no neat faceting or periodic order has been observed [26]. In contrast, Imai et al. showed that the addition of the neutral template F127 to cetyltrimethylammonium bromide (CTAB) leads to well-faceted nanoparticles, which exhibit a somewhat large distribution of sizes and some shape diversity [27,28]. The nonionic triblock co-polymer F127 is considered here as a growth suppressant. The inutility of dilution and pH control are consistent with the chemical growth control mode of action of both additives. There is one example of monodispersion attested below 50 nm, combining initial high dilution with the addition of TEA [29]. Despite a much lower quantity of TEA compared to the earlier reports (0.25 instead of 1 mol eq. vs. TEOS), these particles still exhibit a very low degree of ordering, as reported in all the other contributions utilizing TEA additives [21,22,23]. Therefore, the actual size threshold for well-structured mesoporous particles is still represented by the transitional ovoid particles of 50 nm first depicted by Mann et al. in 2002 and can be proposed as the upper limit for ultra-small nanosized mesoporous objects [12]. To our knowledge, scarce examples of structured ultra-small mesoporous silica nanoparticles (US-MSNs) have been observed in polydisperse systems, and there is only one claim of monodispersion, without any details about the synthetic process or a detailed investigation of their structural state [30].

Here, we address both issues, showing that the nucleation and growth quenching sequences using dilution and pH control in the presence of a tiny amount of nonionic surfactant lead to the monodispersion of US-MSNs quenched in the “on-the-edge” crystallization state. This is documented thanks to a thorough 3D HR-TEM investigation using 2D-image FT transform analysis. Their surprisingly high stability under the beam and calcination prompted us to investigate the porosity and surface area of interest for catalysis and separation applications.

## 2. Materials and Methods

### 2.1. Synthesis of the Nanoparticles

Synthesis of MSN-23-A: The molar ratio of the synthesis solution was 1 SiO_2_:0.5 NaOH:0.12 CTAB:5.2 10^−5^ F127:130 H_2_O. CTAB (Fisher, Bourgoin, France) (0.2 g or 5.5 10^−4^ mol) and F127 (0.003 g or 2.4 10^−7^ mol) were dissolved in a solution of NaOH (1 M or 2.3 g) and distilled H_2_O (8.78 g). After a short time (40 s), TEOS (Alfa Aesar, Haverhill, MA, USA) (1 mL) was added. Dilution by 4, 6, or 8 times was then performed by adding 50, 75, or 100 mL of distilled vwater, respectively. Four minutes later, the pH was adjusted to *ca.* 5.5 using about 1.1 mL of 2 M HCl solution. Finally, 3 h later, NaOH was added to increase the pH up to *ca.* 7. The stirring was stopped after 1 h. The suspension was then kept at RT statically and was found to be stable for more than half a year. A solid was obtained by centrifugation, washing, and re-dispersing in distilled water under ultrasonic treatment three times. Surfactants were removed by calcination under air flow while the temperature was increased from 25 to 550 °C for 6 h and then kept at 550 °C for another 6 h. *Elemental analysis.* MSN-23-A (wt %): C—47.15; N—2.89; H—9.30; and residual mass at 1000 °C—63.

Syntheses of MSN-100-B and MSN-100-A: MSN-100-B was synthesized as described elsewhere and characterized as nanoparticles with different shapes and an average size of 100 nm (TEM image in Figure S6) [27,28]. MSN-100-A was obtained from MSN-100-B upon treatment at a pH of 5.5 for 3 h, followed by neutralization according to the same procedure as the two last steps of the synthesis of MSN-23-A. *Elemental analysis.* MSN-100-B (wt %): C—31.75; N—2.43; H—6.35; and residual mass at 1000 °C from TGA—33.1 and MSN-100-A (wt %): C—26.88; N—1.53; H—5.51; and residual mass at 1000 °C—44.2.

### 2.2. Transmission Electron Microscopy

A drop of diluted MSN colloidal solution was deposited on a carbon grid and dried at room temperature. High-angle annular dark-field (HAADF)-STEM (also known as Z-contrast) images were taken at room temperature using a JEOL JEM-2100F field-emission microscope equipped with a JEOL ADF detector (Tokyo, Japan). The microscope was operated at 200 kV. The probe size and camera length were 0.45 nm and 8 cm, respectively. The regular HR-TEM images were obtained using a JEOL 2010. TEM tilting experiments were carried out, using a JEOL 2010 microscope operated at 200 kV and equipped with a home-made sample holder designed for tilting on a large angular range comprised between −20 and +20° [31].

### 2.3. Image Analysis

Filtered images and diffraction patterns were obtained from direct and inverse 2D Fourier transform combined with ad hoc gray threshold filtering using freely available IMAGEJ™ software. The diffraction pattern by 2D FT of the images needed contrast enhancement and, eventually, contrast inversion, for better observation. In addition, rings between ca. 0.9 and 1.8 nm^−1^ or above in the reciprocal space (diffraction image obtained by FT) occurred due to optical artifacts (picture grain). Gray threshold filtering allowed us to remove these rings, and then, FT-filtered pictures were obtained.

### 2.4. DLS, XR, TGA, and Nitrogen Isotherms

DLS measurements were performed at room temperature using a Cordouan DLS135 instrument and applied to the synthetic solution either in their current state or after dilution and ultrasonication to reach the size of non-aggregated particles [9]. XRD was carried out on both the as-synthesized and calcined samples with a Bruker (Billerica, MA, USA) (D8 Advance) diffractometer using Cu K_α_ monochromatic radiation. TGA was performed on the as-synthesized samples with a Netzsch STA 409 PC/PG instrument under air flow (30 mL min^−1^) with a temperature increase rate of 10 °C min^−1^. Nitrogen sorption isotherms were measured at 77 K on the calcined samples with a Belsorp-max. A pre-treatment process at 350 °C under vacuum was performed before taking sorption measurements.

## 3. Results and Discussion

### 3.1. Time-Controlled Synthesis Approach 

**Figure 1:**
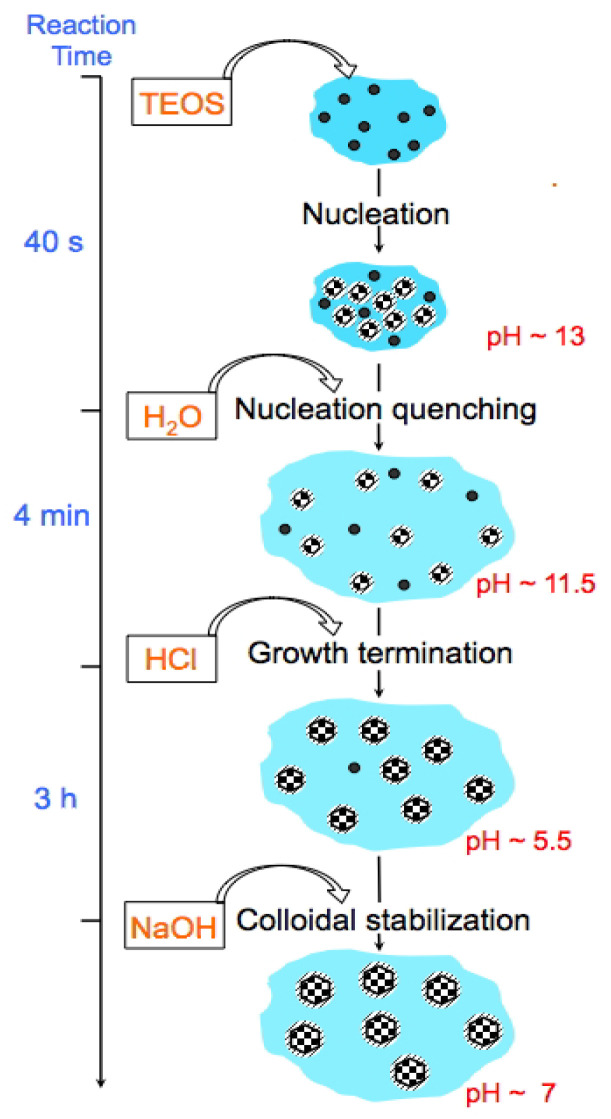
Time-dependent scheme of MSN-23-A synthesis: the black dots depict oily droplets of partially hydrolyzed TEOS, the black and white checkerboard-filled circles are US-MSNs, and the dashed areas around the circles represent the surfactant layers covering the particles.

**Figure 2:**
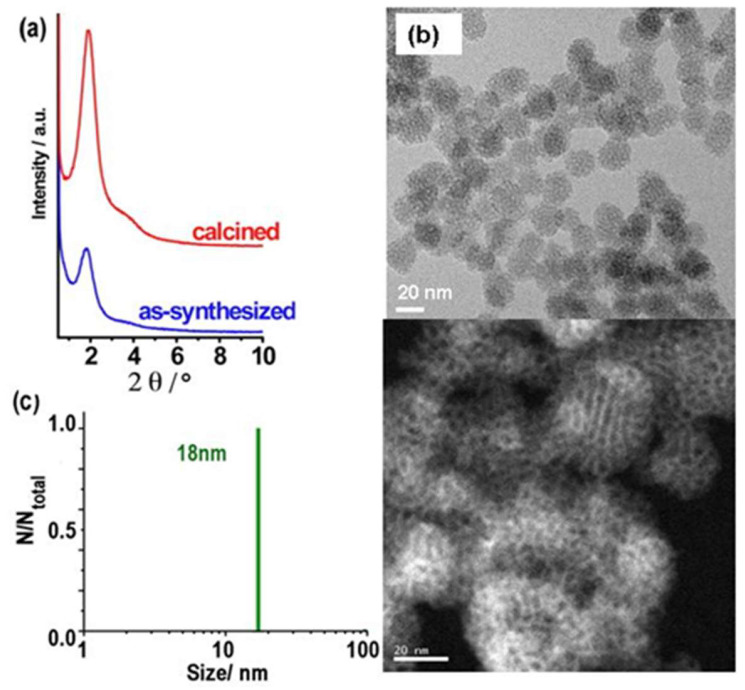
(**a**) XRD patterns, (**b**) HR-TEM images (white bar scales: 20 nm for the bottom with inverted contrast), and (**c**) DLS size distribution of ultrasonicated as-synthesized MSN-23-A nanoparticles. Composition of the synthesis solution: 1 SiO_2_:0.5 NaOH:0.12 CTAB:0.000052 F127:130 H_2_O. Nucleation quenching was performed using dilution by 6 times (CTA:F127 = 2300:1 molar ratio).

The synthesis protocol of the US-MSNs is schematized in Figure 1, where the timing, addition of chemicals, pH values, and reaction steps are provided. Note the additional treatment for 3 h at a pH of 5.5 and the presence of F127 at the beginning of the synthesis, which are novel features in comparison to Mann et al.’s reports [10,11,12] TEOS was added to an aqueous solution (pH~13) containing CTAB and a tiny amount of triblock co-polymer Pluronic F127, i.e., 1.5 (wt)% with a CTAB/F127 molar ratio of 2300. The above reaction mixture was stirred at room temperature for 40 s. Then, a rapid aqueous dilution by six times led to a pH drop to 11.5. After 4 min, the pH was reduced to ~5.5 using HCl, followed by neutralization after 3 h using NaOH and equilibration at a pH of 7 for another hour under stirring. The final colloidal solution was stocked as such at room temperature and found to be stable for at least 6 months. The as-synthesized mesoporous silica nanoparticles (23 ± 3 nm, Figure 2b, vide infra) using this acidic treatment before neutralization are referred as to MSN-23-A. A white powder obtained by centrifugation (11,000 rpm) was washed three times with distilled water and eventually calcined under air flow at 550 °C to remove the organic surfactants (see Section 2.1). For comparison, another set of nanoparticles was prepared according to the acidic route (pH 0.5, instead of basic) and dual templating technique reported by Imai et al., which does not use a quenching sequence and yields an average particle size of 100 nm; the sample is referred to as MSN-100-B [27,28]. In the latter case, the TEOS concentration was close to that used in the synthesis of MSN-23-A, producing about five times more CTA^+^ and fifty times more F127.

### 3.2. Structure and Morphology 

The XRD diagrams of calcined MSN-23-A exhibited a strong peak at a *d* spacing of 4.9 ± 0.1 nm, which was much more intense than all the previous reports concerning nanoparticles in the size range of 20 to 50 nm (see introduction). A single weak and broad shoulder on the high-angle side covering the range where secondary peaks were expected for hexagonal or cubic MCM-41 or MCM-48 types of structure precludes any definitive structural assignment (Figure 2a). High-resolution transmission electron microscopy (HR-TEM) and scanning electron microscopy (SEM) images showed that both the as-synthesized and calcined MSN-23-A samples contained nanoparticles with a narrow size distribution in the range of 18 to 25 nm, which were rather faceted (Figure 2 and Figure S1, respectively).

Close xA examination of their shapes revealed that the angles between facets ranged from 100 to 150°. Despite the large number of US-MSNs observed for each sample (>200), rectangular or square shapes were too scarce or too difficult to observe, and the typical 2D hexagonal array of channels was not observed, discarding regular 2D hexagonal or cubic arrays. Although such a structure is clearly observed for nanoparticles or fibers as small as 60 to 100 nm [10,11,12,13,32], further investigations are necessary to clarify this point. Indeed, distorted channels with various angles of emergence from the particles could blur the observation. Alternatively, the preferential orientation of the particles on the TEM grid could complicate the observations.

**Figure 3:**
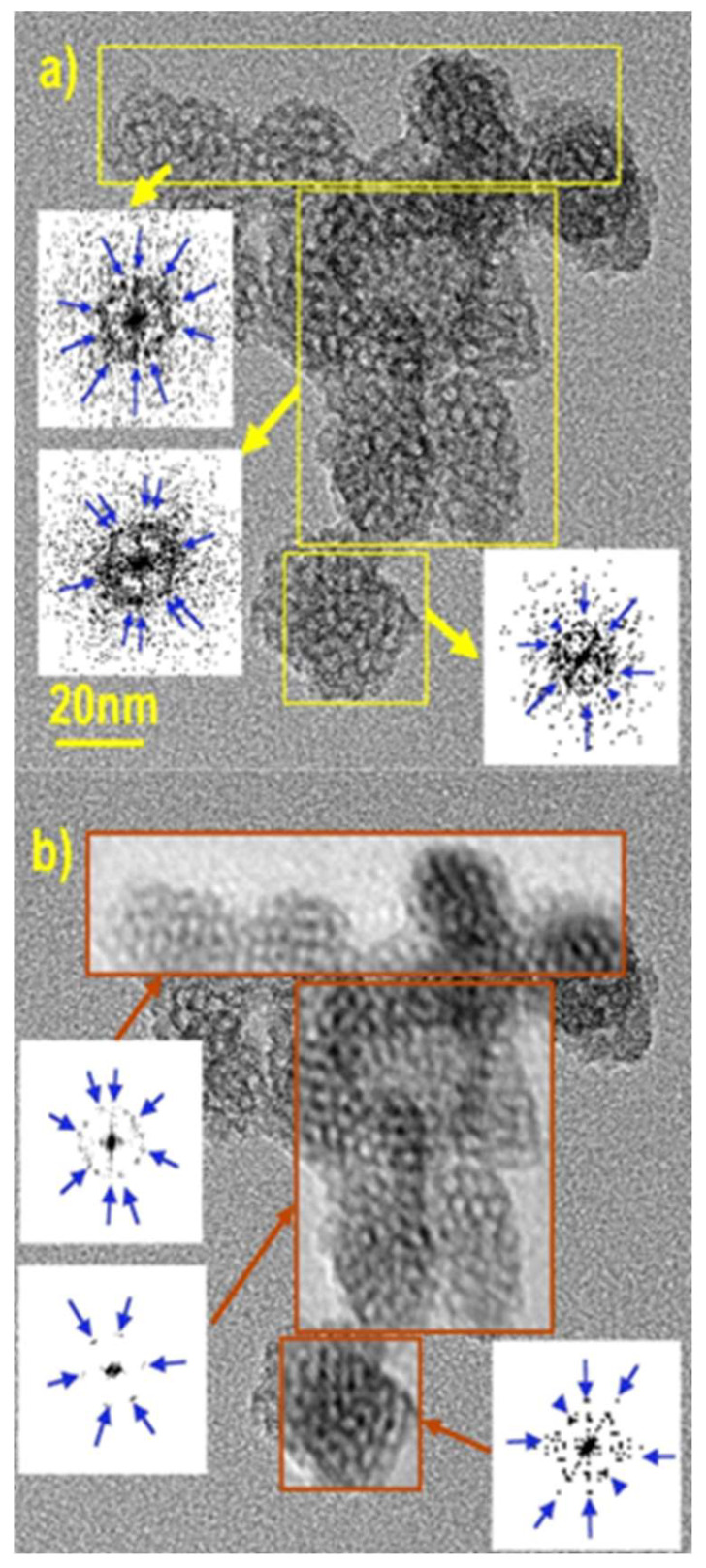
HR-TEM image of calcined MSN-23-A on a typical cluster of US-MSN particles: (**a**) areas selected for 2D FT transform, producing diffraction patterns represented in reverse contrast (yellow arrows connect the FT transformed area to the corresponding diffraction pattern, and blue arrows are provided on the pattern as a guide) and (**b**) the same TEM image with the same area as in (**a**) replaced by their back FT transform (red-brown arrows connect the gray threshold filtrated diffraction pattern with the corresponding back FT transformed area (the blue arrows have the same function as in (**a**)).

To further document this point, diffraction patterns and FT-filtered images of the calcined samples were analyzed thoroughly. Indeed, with the channels being empty, better-contrasted images of the internal structure were obtained. For instance, Figure 3 depicts a cluster of 20- to 30-nm particles with periodically contrasted bodies, consistent with internal pores of nearly the same size, i.e., about 3 nm large. The three selected areas (yellow frames) also exhibit complex diffraction patterns dominated by dots forming rings, consistent with a mixture of monodomains (the blue arrows highlight some of the most contrasted dots). This shows that the neighboring particles did not share the same direction of ordering. In the center of the cluster in Figure 3a, the ordered domains seemed more extended but still belonged to two different hexagonal crystals, the orientation of which differed from each other by *ca.* 10°. A gray threshold filtering on these diffraction patterns afforded a simplified picture that still kept the most important features and led to the same conclusion, i.e., the presence of two different hexagonal domains tilted by 10° (Figure 3b). The hexagonal diffracting patterns exhibited dots at reciprocal distances of 0.258 ± 0.004 nm^−1^, i.e., 3.9 nm, accounting for *a*_0_ = 4.5 nm. This was apparently at odds with the XRD peak at 4.9 nm, which would correspond to a channel-to-channel distance of 4.9 or 5.7 nm for a cubic array or a hexagonal one. Alternatively, when enough pores were aligned with each other, an intensity profile analysis of their TEM images exhibited periodicities of 5.3 ± 0.1 nm only in between distances calculated assuming simple cubic or hexagonal arrays. These apparent discrepancies were assigned to a mixture of symmetries and/or an absence of a medium-range order inside the nanoparticles. In addition, there were obvious defects in all the areas selected. In particular, the particle lying at the bottom of Figure 3 was characterized by a step defect crossing at 45° through its structure. A survey of other regions confirmed that the pure hexagonal array was not the dominating feature.

**Figure 4:**
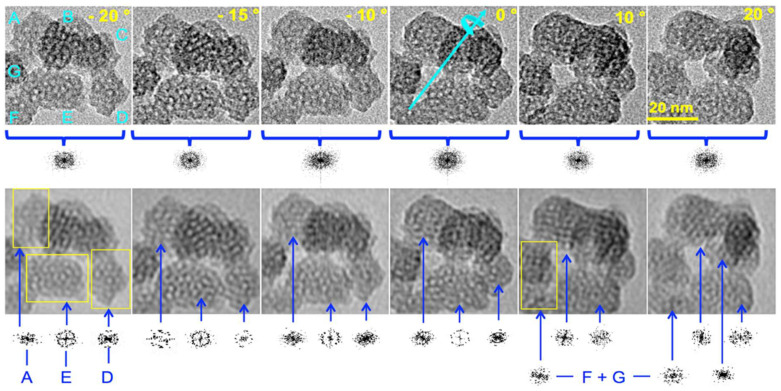
HR-TEM image of a typical cluster of nanoparticles of calcined MSN-23-A at different tilt angles: rough images (**top**), corresponding 2D FT images (**bottom**), tilt angles (top right of each image in the top row) with the same scale (top right image), diffraction pattern provided below each image, total FT (top row), and individual particle diffraction (bottom row) for only A, E, and D on the first four images, avoiding superimposition and the moiré effect (see text). F and G, taken together, as well as the A and E diffraction patterns are provided in the two last images on the bottom right, but the diffraction pattern for D is not shown because of the superimposition with particle B. Note that in the last image (+20 °C), particle C, which is pointed by the longest blue arrow, emerges between particles A and B and above E.

To obtain deeper insights into the intimate ordering of the particles and to address the question of preferential orientation or unusual symmetry, HR-TEM images of particles were taken at different incident angles by tilting the sample under the beam from −20 to +20° (Figure 4). At first sight, there was no obvious apparition of parallel channels while most of the particles looked roughly alike, consistent with no preferential orientation. Another advantage of tilting was the change in the contrast between pore walls and pore voids. This was clearly seen on particles A, D, and E. By contrast, particles B and C, as well as F and G, were superimposed onto one another, generating a moiré pattern that prevented any simple interpretation in terms of structure. The white spots in particles A, D, and E, corresponding to pores aligned with the direction of observation, appeared with different levels of contrast. Their diameters also appeared smaller than the diameter measured from the N_2_ adsorption–desorption curves (1 to 2 nm instead of 3.9 nm, vide infra). The channel diameter appeared to be smaller than it should, which is usually attributed to channel curvature in the bulky MCM41 [33,34]. Indeed, short-range curvature characterized on large ovoid MCM-41 nanoparticles (>50 nm) could be the rationale for this, although it was inconsistent with the presence of facets and an intense XRD peak [12]. A radial array of channels generated in the presence of TEA molecules was also discarded for the same reasons [16,22,23]. This suggests that the pores were connected to one another through restricted apertures.

Furthermore, Figure 4 shows that the cluster of particles at different tilted angles generated a diffraction pattern consistent with different domains that are ill-oriented with one another. Therefore, each particle was investigated individually by 2D FT analysis using the region of the image delimited by yellow frames on the FT-filtered image. Individual diffraction patterns were obtained for each portion of the image included in the frame. In the top left corner, particle A exhibited the most faceted aspect among US-MSNs of this cluster, and its diffraction pattern depended on the tilt angle, i.e., ill-defined at −20 and 0°, orthorhombic at −15 and +10°, close to hexagonal at −10°, and 1D-ordered (with two opposite diffraction points) at +20°. This is typical of 3D ordering, with some diffraction images that are much better oriented than others. On the top left of the 2D FT-filtered image taken at +10°, there was a definitive cubic array with a step defect on the right side. The rationale for this is a mixture of faulted hexagonal and cubic symmetries in the same nanoparticle. In the bottom center of the image, particle E exhibited a diffraction pattern with an overall fourfold symmetry axis at −20° tilt and a sixfold symmetry at 0° tilts. Strikingly, at −20°, particle E, which had the least faceted appearance, possessed a circular array of pores on the right side and a faulted hexagonal symmetry on the left side. There was also an obvious fivefold array in the center of the particle. At a tilt of −15°, the diffraction seemed to be based on a hexagonal pattern awkwardly split into a four-leaf clover pattern and a top side with four faces of half of an octagon. Particle D, on the bottom right of each image, exhibited a 90° clockwise-rotated muffin-like shape at −20°, the base of which had the shape of half of a hexagon. The corresponding diffraction pattern definitively showed an order, although it was complicated to assign. Both particles F and G presented more faulted structures than other particles. On particle C, which emerged between particles A and B, parallel diffracting lines appeared at a tilt of +20° (center right image, Figure 4). This was a rare example, which could account for a transversal view of a 1D hexagonal array of channels expected in an MCM-41 mesophase. A large survey showed that the particles of Figure 4 were much more representative than those of Figure 3. Therefore, particles characterized by well-defined short-range order and ill-defined medium-range order were the dominant features (Figure 4), while “good-looking” particles with a quasi-hexagonal close-packing array represented rather rare instances (Figure 3). All these particles were prefiguring cubic or hexagonal arrays of pores but had not yet reached this level of ordering.

As a complement to TEM, the dynamic light scattering (DLS) study provided the hydrodynamic diameters of the particles or their aggregates, moving coherently with some solvent and surfactant molecules [9]. In the synthetic solution, the hydrodynamic diameters were distributed between 24 and 41 nm, accounting for both isolated and aggregated nanoparticles (Figure S3a). In fact, after dilution and ultrasonication treatment, the flocculates were dismantled, giving rise to monodispersed particles characterized as a single DLS peak at 18 ± 2 nm. This confirms the very narrow distribution of sizes centered at a size slightly smaller than those characterized by HR-TEM (23 ± 3 nm) (Figure 2c). Aggregates were indeed seen in TEM images as the superimposition of small primary particles. These fragile aggregates or nanoflocculates were themselves small enough to yield stable colloidal solutions at room temperature (RT) for several months. After more than half a year at a pH of ~6.5 to 7, the average size of the flocculates increased slightly, yielding a distribution ranging from 37 to 70 nm (Figure S3b), although the primary particle size was unchanged according to HR-TEM observation. This would correspond to *ca.* 3 to 12 or 8 to 60 US-MSNs per flocculate in fresh solution or in 3-month aged solution, respectively. This slow flocculation allowed the harvesting of more particles by centrifugation after a longer period of time. For instance, the silica yield was close to a low percentage of fresh solution and *ca.* 60% after one month. Note that both CTA^+^ and silica yields were identical as the CTA^+^/SiO_2_ molar ratio was the same in both US-MSNs and the synthesis solution (see Section 2.1).

### 3.3. Structural and Textural Porosity 

**Figure 5:**
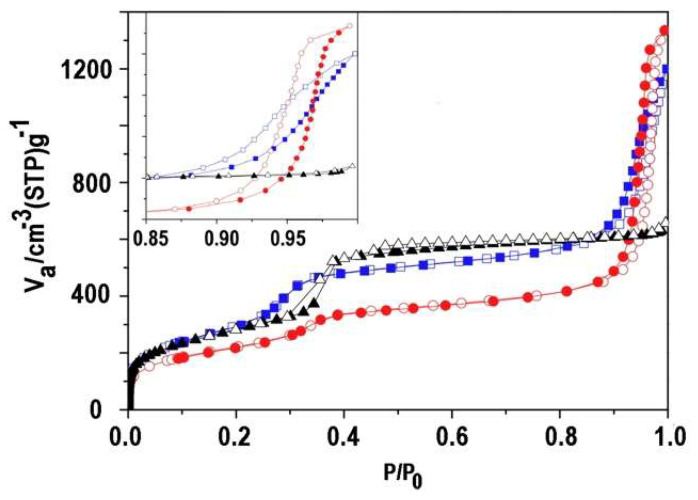
N_2_ adsorption–desorption isotherms of MSN-23-A (red round mark), MSN-100-B (blue square mark), and bulk classical MCM-41 (black triangle mark). Insert: enlarged part from 0.85 to 1.0 P/P_0_; adsorption (full symbol); and desorption (empty symbol).

The nitrogen adsorption–desorption profile of the calcined ultra-small nanoparticles MSN-23-A was compared to those of MSN-100-B and bulk MCM-41 (Figure 5 and Table S2). Both types of nanoparticles presented a steep step in the reduced pressure range, i.e., *P*/*P*_0_ = 0.27–0.34, which is typical of the capillary condensation in a templated internal mesoporous network of 3.9 nm diameter for MSN-23-A and bulk MCM-41 and 3.5 nm diameter for MSN-100-B according to the BdB method (D_BDB_) [35,36]. Note that the size distribution for MSN-23-A was ±0.2 nm and twice as large as MSN-100-B and bulk MCM-41 [13,14,27,28,35,36,37]. 

The height of the adsorption plateau taken before a second hysteresis loop at *P*/*P*_0_ = 0.8 was similar for MSN-100-B and bulk MCM-41 and represented internal pore volumes of 0.83 and 0.88 ± 0.02 cm^3^ g^−1^, while it was smaller for MSN-23-A, at 0.63 ± 0.02 cm^3^ g^−1^. The pore wall thickness calculated from XRD and pore sizes was similar for MSN-100-B and bulk MCM-41, i.e., 0.9 and 1.0 ± 0.2 nm, respectively, and much larger for MSN-23-A, i.e., 1.8 ± 0.3 nm, assuming a hexagonal array. Note that TEM contrast profile analysis provides a smaller estimate of 1.4 ± 0.2 nm for the latter.

Assuming the same pore wall density as for bulk MCM-41 and considering the pore volume of the latter as a reference, the internal pore volume would vary as the ratio of pore diameter to pore wall thickness, i.e., 0.49 (from XRD and adsorption) or 0.63 (wall thickness from TEM contrast analysis) and 0.88 cm^3^/g for MSN-23-A and MSN-100-B, respectively. The best wall thickness estimate of 1.4 nm was obtained from TEM. This smaller estimate was still 40% larger than in MSN-100-B and in bulk MCM-41 materials. Indeed, the pore walls of MCM-41 are known to contain asperities large enough to fit argon atoms, similar to MSN-100-B [38]. Accordingly, MSN-23-A might possess fewer or smaller asperities than that of the other materials.

The vertical hysteresis loops at high relative pressure seen only for nanoparticles were assigned to interparticle void (Figure 5, insert). Indeed, the absence of such a loop in bulk MCM-41 indicated that the voids between grains or fibers were too large (>200 nm) for N_2_ adsorption–desorption measurements. In MSN-100-B and MSN-23-A, their sizes and volumes calculated on the adsorption branch were 38 and 68 nm for 0.94 ± 0.01 and 1.41 ± 0.02 cm^3^ g^−1^, respectively. In the former, the voids were smaller than the particle size, indicating a rather efficient packing density. In contrast, MSN-23-A exhibited larger void sizes than the particle size, consistent with a rather low packing density for an extremely large unoccupied volume. Narrow void size distribution and a high volume of about 1 cm^3^ g^−1^ have also been reported in packed monodispersed 90 nm zeolite [9].

### 3.4. Thermal and Hydrothermal Stability 

Similar to bulk MCM-41, the peak intensity of the XRD diffraction pattern after surfactant subtraction was twice as intense, with no noticeable broadening, consistent with full retention of structure and a mere replacement of the surfactant by air and some adsorbed water coming from ambient humidity (Figure 2a) [37]. Moreover, the absence of shift of the main XRD peak after calcination indicated that MSN-23-A did not undergo any shrinkage up to 550 °C in air, contrary to most of the reported bulk materials [14,15]. The US-MSNs that were stable for several months in water in the presence of the surfactant were also subjected to stability tests in distilled water overnight after the removal of the surfactant by calcination. MSN-23-A showed remarkable stability towards shrinkage (no XRD peak shift) and little loss of ordering (little intensity loss for the same peak width, Figure S2). By contrast, calcined MSN-100-B exhibited a significant shrinkage and a drastic loss of ordering (intensity loss and shift towards high diffraction angles). This was a strong indication that the polycondensation of the siliceous wall was terminated at room temperature and led to a highly stable structure. This corroborated the above hypothesis for thicker and/or denser pore walls. Unfortunately, the pore wall densification was not related to any change in the ratio of the Q3 to Q4 signal on the solid-state ^29^Si NMR signal, which was particularly large for MSN-23-A (not reported here), or for MCM-41 obtained by fast microwave at 200 °C with narrow pore walls (0.9 nm) and a very high degree of ordering (narrow XRD peaks with no shrinkage upon calcination) [39].

### 3.5. Surfactant-to-Silica Balance in US-MSNs 

The amount of surfactant retained in the as-synthesized nanoparticles was estimated from TGA (Figure S2). Below 150 °C, the mass loss was due to water desorption (2 to 3% of the total mass loss). Above 150 °C, the mass loss was assigned to surfactant oxidation [40]. In the latter range of temperatures, a much larger mass loss was measured for MSN-100-B (67 ± 2%) than for MSN-23-A (37 ± 1%) or for bulk MCM-41 (40 ± 1 wt%, accounting for *ca.* 0.17 surfactant/Si molar ratio in the latter case) [14,36]. Taking bulk MCM-41 as a reference and assuming an equivalent pore-filling factor by the surfactant in all three materials, the mass losses should be proportional to internal pore volumes. Accordingly, from the N_2_ physisorption data, these volumes would be *ca.* 35 wt% in MSN-23-A and only 42 wt% in MSN-100-B, accounting for 0.12 and 0.16 surfactant/Si mol ratios, respectively. The 25 wt% excess of mass loss in MSN-100-B might be due to the presence of a much higher loading in secondary surfactant F127 (70 wt% in MSN-100-B and 1.5 wt% in MSN-23-A) [22,27,41]. However, this was at odds in both solids, with the elemental analysis revealing a C/N ratio very close to 19, which mostly characterizes CTA^+^ cations, discarding the presence of F127 in the nanoparticles after washing the powder with distilled water. Then, it would be tempting to assign the strong surfactant retention to the smaller size of the interparticle voids in MSN-100-B (38 nm) than in MSN-23-A (68 nm) [42]. However, the synthesis of MSN-100-B resulted in a very acidic media, apparently favorable for high cationic surfactant retention since washing with water at a pH of 5.5–7 eliminated this excess of surfactant (Figure S2). Then, nanoparticles of MSN-100-B and bulk MCM-41 were alike in terms of internal pore filling with the surfactant, while MSN-23-A contained fewer surfactant molecules, in agreement with thicker or denser pore walls.

### 3.6. Synthesis Parameters versus Particle Size, Morphology, and Colloidal Stability 

The best results were obtained with an Si:CTA^+^:F127 molar ratio of 19,200:2300:1, which was used for MSN-23-A. The nanoparticles exhibited a positive zeta potential evolving with time, from +24 to +32 mV at a pH of 5.5, which was not high enough to avoid flocculation for long-time storage at this pH (Table S1). Thus, an adjustment to pH 7 was necessary after 3 h, during which the zeta potential increased progressively from +42 to +59 mV, leading to the colloidal solution being stable for several months (Figure S3). Positive potentials were consistent with a double layer of cationic surfactant covering the negatively charged silica surface [29]. The increase in potential after neutralization by the addition of NaOH could be related to Na^+^ adsorption. However, this element was not detected by EDX. The rationale was rather a higher CTA^+^ coverage favored by a higher density of surface silanolate groups (SiO^−^) generated at a higher pH.

**Figure 6:**
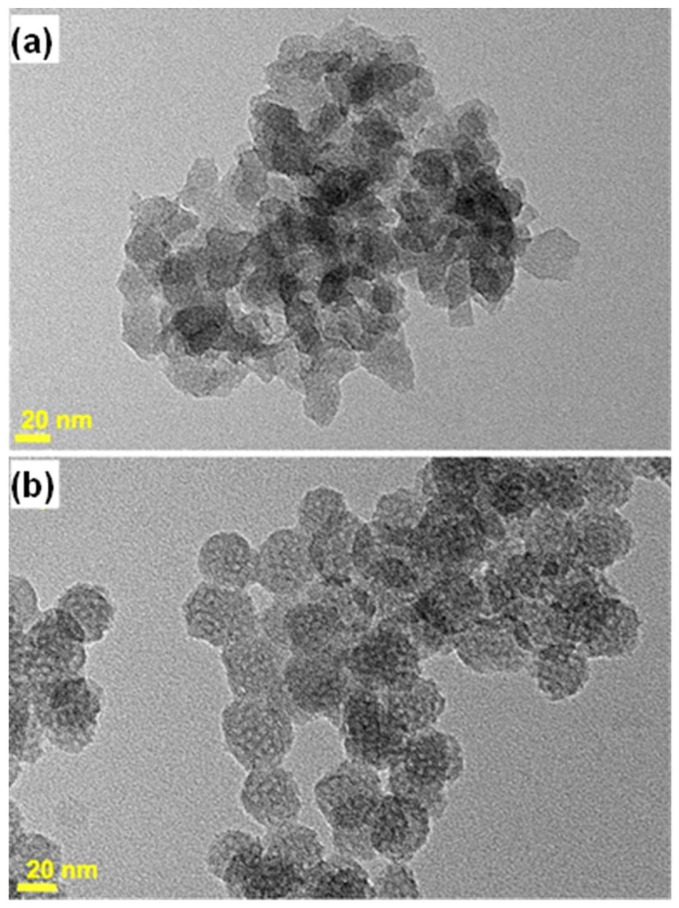
TEM images of MSN-18-A and MSN-30-A obtained with the same surfactant molar ratio as in the synthesis of MSN-23-A, with a dilution of 4 (**a**) and 8 (**b**) instead of 6, as shown in Figure 1.

The effect of dilution on nucleation quenching is illustrated in Figure 6. For a dilution by four (MSN-18-A) instead of six times, the average size was similar (~20 nm), but the size distribution was wider (15 to 30 nm), and the shapes were more heterogeneous (Figure 6a). The channels were also more difficult to observe in the TEM images, suggesting a less ordered structure. In addition, these particles aggregated into large and compact domains. These differences in comparison to the best samples were assigned to an inefficient nucleation quenching, keeping supersaturation conditions at work for secondary nucleations to proceed, thus broadening the distribution of sizes. For a dilution by eight times, the nanoparticles became larger, ranging from 25 to 40 nm, consistent with a lower number of nuclei (MSN-30-A, Figure 6b). Therefore, the nucleation blockage seemed likely to be achieved, while a larger distribution of sizes and shapes suggested that the nuclei were unstable and underwent Oswald ripening.

The effect of varying the proportions of surfactants between them and in relation to the silicon precursors was also investigated. With twice more F127 (2300 CTA^+^ to 2 F127), heterogeneous particle shapes and sizes were produced, some rather small (~25 nm) and others much larger (up to ~100 nm), including ellipsoidal particles of 40 to 60 nm (Figure S4a). The latter resembled those described previously in the absence of F127 [12]. For twice less F127 (2300 CTA^+^:0.5 F127), small faceted particles of about 17 nm, presenting various shapes, were mixed with larger ones of about 20–25 nm (Figure S4b). This might be due to a lack of secondary surfactant that covers each particle inappropriately. When CTA^+^ concentration was doubled, i.e., 4600 CTA^+^:1 F127, larger sizes were observed in the range of 18 to 40 nm (Figure S4c). In addition, the particles exhibited irregular shapes, from ill-faceted to non-spheroid. This was likely due to the merging of primary particles and/or a face-dependent growth rate. On the contrary, decreasing the CTA^+^ concentration but not that of F127 (1150 CTA^+^:1 F127) two times yielded relatively well-structured small particles. These conditions corresponded to twice less surfactant of both kinds than in the first modification proposed above (Figure S4d). However, more particle merging took place, and particle isolation by ultra-centrifugation was more difficult to achieve. The rationale behind these experiments was the control of the nucleation rate by CTA^+^ and its quenching by dilution, while the neutral F127 minimized the coalescence of particles with one another.

Indeed, a higher concentration of CTA^+^ led to smaller particles due to higher supersaturation conditions, which led to a larger number of nuclei and, therefore, to smaller particles. Then, a dilution that was too small produced a larger size distribution with ill-blocked nucleation conditions, while a high concentration led to merging. The latter effect seemed to be better controlled, with Oswald ripening in the presence of F127, the latter being likely adsorbed on the US-MSN surface. Note that an oversaturation of F127 was detrimental to the particle size distribution, showing that F127 should be restrained to its role on the surface and should not interfere with CTA^+^ for both the nucleation rate and internal pore structuring. Indeed, in MSN-100-B, a smaller pore size than in bulk MCM-41 or in the present particles revealed the presence of both surfactants in the internal structure. The optimum CTA^+^/F127 ratio proposed here was found by trial and error since it was not possible to detect its presence on the surface of the US-MSN.

**Figure 7:**
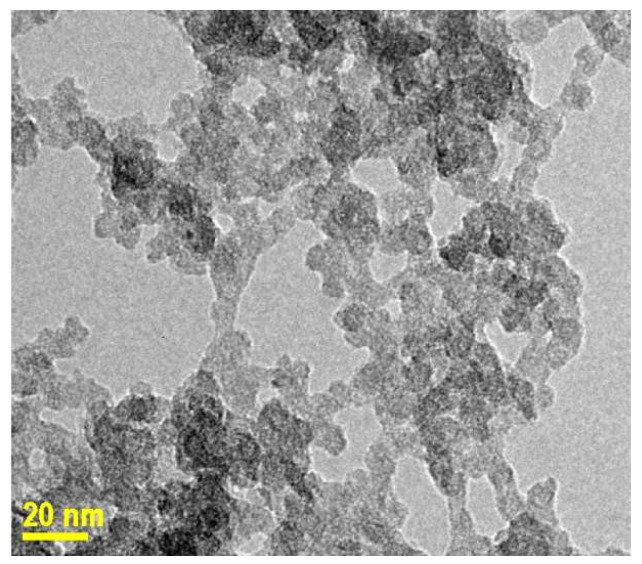
TEM image of MSN-23-A at a pH of 5.5 before the neutralization step.

pH control was also decisive for the formation of well-structured particles. In fact, during the treatment at pH = 5.5 for 3 h, small oily droplets of TEOS were still present and progressively disappeared. In this sequence, the system was still in a slow-growth regime where particle merging or coalescence could take place without the dual control by CTA^+^ and F127. Indeed, TEM images showed that the particles had not yet reached either the final internal ordering or their final sizes (7 to 10 nm) (Figure 7). Their smooth surface and the absence of visible pores suggested that they were covered by an amorphous layer of silica precursor that might contain partly hydrolyzed TEOS and presented some analogies with the immature nanoblocks observed during the induction period of a zeolite growth [9]. Contrary to zeolitic nanoblocks, the actual US-MSNs were not crystalline and cannot be considered as nuclei stricto sensu since they did not present any defined structure found in bulk MCM-41 or -48 materials. The second difference with the zeolitic growth process is a smaller synthesis temperature of 25° C instead of 80 °C. The nanoblock aggregation into larger particles of *ca*. 90 nm was avoided here, probably using higher dilution and acidic quenching. This is consistent with previous studies showing that nanoparticles are preferentially generated between a pH of 3 and 6 using CTA^+^ alone [43].

### 3.7. Role of the F127 Additive as a Charge Density Modulator and Particle Size Stabilizer 

In contrast to all previous works using large quantities of additives, here, very small amounts of F127 sufficed (typically 1.5 wt%, with an F127/Si molar ratio of 1 to 2300). It was previously stated that the neutral surfactant acts as a growth suppressant by fully covering the particle’s external surface [27,28]. In the present case, there was not enough F127 to cover the external surface of the particles. The positive zeta potential showed that the surface was covered by the CTA^+^ surfactant, in agreement with previous observations [23,44]. Then, F127 was likely to intervene in this CTA^+^ layer since it prevented the coalescence of the US-MSN. 

**Figure 8:**
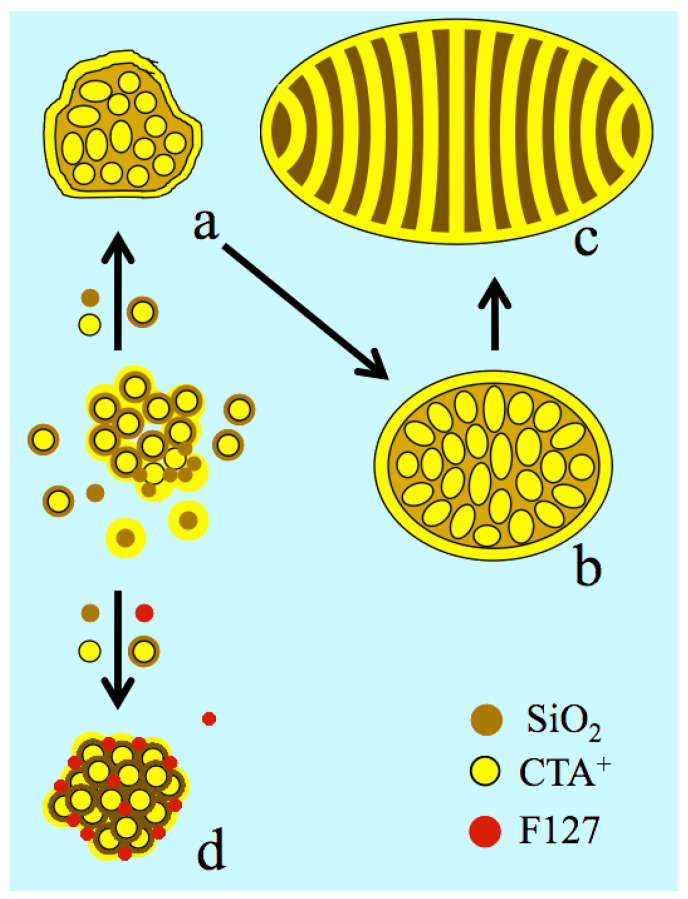
Nanoparticle morphologies produced from silica–micelle hybrids, including silica-coated micelles according to the F127 free route ((**a**–**c**): ref. [12]) and in the presence of F127, including the aggregation, ordering, and stabilization of the silica-coated packing (**d**). CTA^+^ is shown in yellow, the circles for spherical micelles or strips for surfactant double layers around the micelles and F127 are shown in red, and light and dark brown colors represent the silicate oligomer precursors and polymerized silica, respectively. (**a**) Ill-ordered strawberry-like particle, (**b**) intermediate state of nanoparticles, implying the elongation of micelles and pre-ordering, (**c**) ovoid particles (~50, see Figure S4a), and (**d**) US-MSN produced from a 3D array of nano-hollow spheres in the presence of F127.

Indeed, one expects the formation of an {S^+^, I^−^} interface on the external surface, like in the pores, where S^+^ is the cationic surfactant and I^−^ the inorganic surface. The internal curvature that drives the control on such a type of mesophase (lamellar, hexagonal, or cubic) is obtained from the surfactant-to-silicon ratio or from the synthesis temperature, which affects the condensation of silica and silanol density [45,46,47,48]. A perfect match of charge density yields flat surfaces, while a mismatch generates convex or concave surfaces depending on which side the charge density is the highest (Figure S5) [49]. Since the surfactant is also adsorbed on the external surface, it affects also the particle morphology and size [43,49]. Thus, it is likely that F127 was modulating the external charge density, releasing the surface tension of the US-MSN. Such a role has been reported for the surfactant counter ions, X^−^, which intervene directly in the double electrical interface, represented as {S^+^, *m*X^−^, (1 − *m*)I^−^} [49,50,51,52]. Changing the cationic head group or intercalating anionic surfactant also produces a curvature control [53,54]. Since neutral organic molecules and neutral alkyl-polyethylene block co-polymers favor the formation of MCM-48 cubic mesophase [55,56], they likely act on charge density matching. F127, which is an ethyleneoxide-propyleneoxide EO-PO-EO triblock co-polymer, is likely to behave similarly, stabilizing the nanoparticles and preventing their coalescence (Figure 8).

### 3.8. Protocrystalline US-MSNs as the Precursor State of Nuclei 

The actual nanoparticles were clearly connected to the “raspberry-like” particles that appeared at the very beginning of the growth process, just before the formation of the channel in the ovoid particles (Figure 8) [11,12]. The difference lay in the faceting appearing together with a higher degree of ordering attributed to the presence of F127. Both “raspberry-like” particles and the actual protocrystalline US-MSN likely shared a common genesis from the aggregation of silica-coated micelles, leading to larger pore walls and a smaller specific surface area than in MCM-41 or MCM-48 [12]. In addition, the short-range order in the packed silica-coated micelles clearly indicated that the US-MSN structure solidified before any external forces, like surface tension, drove any further ordering. Note that the soft matter transition avoided here was reported at a much larger scale of sizes for nascent SBA-15 and SBA-16 nuclei [18,19]. Thus, the diversity of arrays within each US-MSN and among them corresponded to a mixture of nascent particles at different stages of maturity, the evolution of which was quenched in the presence of the stabilizing effect of F127. They could be considered as nano-quasicrystal nuclei with respect to the presence of some characteristic non-periodical pentagonal or octagonal arrays, although they do not develop their nascent structure all through their volume [57]. Paracrystallinity is another state of matter related to materials presenting short- and medium-range order, with a lack of long-range order, where crystallized nuclei are embedded in an amorphous matter [58]. This describes the transitional states between amorphous and crystalline phases in vapodeposited thin films of germanium or silicon semiconductors, silica natural deposits (petrification), opals, and diatom earth deposits [59,60,61,62,63]. However, most of the US-MSNs, typically those represented in Figure 4, exhibit a lower degree of structural maturation that is better described as protocystalline. To our knowledge, this term was introduced by Collins et al. in 1998 to describe an “on-the-edge” state before crystallization in vapodeposited thin films of amorphous hydrogenated silicon, exhibiting high performance for solar cell applications [64,65]. The mixture of Si-Si and Si-H bonds creates voids and stabilizes the amorphous state, together with the crystalline nanophase. This structural mixture is the so-called paracrystalline regime [65,66,67]. There is a parallel with the chemistry of porous silica, possessing SiOH groups at the pore interfaces, where the spatial ordering is facilitated by large particles and small pores. This is documented by a large number of reports dealing with micro-, meso-, and macroporous materials [18,19,68,69,70,71].

The actual US-MSNs represent the first isolation of the on-the-edge state of ordering in porous nanoparticles, hence considered as nano-protocrystals, which are soft in solution but can be quenched upon drying and eventually strengthen under calcination or under the electron beam during TEM observation. Their colloidal solutions may be useful for the synthesis of novel materials and structures such as hierarchical porous powder materials, as shown here, or coatings on specific substrates.

## 4. Conclusions

In the present study, a series of monodispersed US-MSNs smaller than 50 nm was synthesized, with a good yield, at room temperature using a modified cationic templated route, to which a time-controlled sequence of dilution and pH adjustments were applied in the presence of a minute amount of F127 neutral surfactant. The slightly acidic treatment allowed nucleation quenching for better size control, while the final neutralization step improved the colloidal stabilization for at least 6 months. F127 was assumed to play a complementary role as a charge density modulator at the external surface, preventing the coalescence of particles. HR-TEM observations at different tilt angles and a thorough analysis of the images clearly demonstrate that the US-MSN particles are on the crystallization edge characterized by partial short-range order, typical of the protocrystalline state. After calcination, the powder exhibited a well-defined hierarchical dual porosity in the mesoporous range (internal pore ~4 nm) (interparticle voids ~70 nm). The US-MSN-23 protocrystals investigated here present a high potential for support not only for separation and catalysis but also for medical applications as nanocarriers in their colloidal state. From this perspective, their capacity for pore size tuning and surface modification remains to be studied. 

## Data Availability

Data are contained within the article.

## Supplementary Materials

The following are available online at https://www.mdpi.com/article/10.3390/nano14121052/s1, Table S1: Zeta potential analysis, Figure S1: HR-TEM image of MSN-23-A, Figure S2: XRD patterns and TGA, Figure S3: DLS size distribution of MSN-20-A, Figure S4: TEM images of US-MSNs, Figure S5: Scheme of charge density mismatch principle, and Figure S6: TEM image of MSN-100.
