# Supplementary material for: Protocrystallinity of Monodispersed Ultra-Small Templated Mesoporous Silica Nanoparticles
Source: Nanomaterials (Basel). 2024 Jun 19;14(12):1052. doi: 10.3390/nano14121052 (PMC11206292; doi:10.3390/nano14121052)
Supplement: Supplementary file 1 [file nanomaterials-14-01052-s001.zip › nanomaterials-3014774-supplementary.pdf]

Supplementary Information

Protocrystallinity of Monodispersed Ultra-Small Templated Mesoporous Silica Nanoparticles

Laurent Bonneviot [1,*], Belén Albela [1], Feifei Gao [1], Pascal Perriat [2], Thierry Epicier [2] and Mohamad El Eter [3]

[1] Laboratoire de Chimie, Ecole Normale Supérieure de Lyon, Université de Lyon, 69364 Lyon, France; belen.albela@ens-lyon.fr (B.A.); feifei.gao@ens-lyon.fr (F.G.)
[2] Matériaux—Ingénierie et Sciences (MATEIS) Unité Mixte de Recherche CNRS, INSA de Lyon, 69621 Villeurbanne, France; pascal.perriat@univ-lyon1.fr (P.P.); thierry.epicier@univ-lyon1.fr (T.E.)
[3] College of Arts and Sciences, American University of Irak, Bagdad, Iraq; mohamad_eter@hotmail.com
* Correspondence: laurent.bonneviot@ens-lyon.fr

Table S1. Zeta potential of the reaction solution for ultrasmall MCM-41nanoparticles, **MSN-20-A**.

Reaction time	Zeta (mV)
(pH 5.5) 5 min	+ 24
(pH 5.5) 4 h	+ 32
(pH 5.5) 3 d	+ 24
(pH 5.5) 4 h → (pH 7) 5 min	+ 42
(pH 5.5) 4 h → (pH 7) 3 d	+ 59

Table S2. Porosity characteristics.

Material	S_{BET} (m^2/g)	Pore diameter[a] (nm)	Pore wall thickness[b] (nm)	Internal Pore volume[c] (cm^3/g)	Interparticle Pore volume[c] (cm^3/g)
MSN-23-A	540 ± 20	3,9 ± 0,2	1.8 ± 0,3	0.60 ± 0.02	1.20 ± 0.02
MSN-20	804 ± 30	3.8 ± 0.2	1.2 ± 0,3	0.63 ± 0.02	1,41 ± 0.02
MSN-100-B	920 ± 30	3.5 ± 0,2	n.a.	0.82 ± n.0.02	0.90 ± 0.02
MCM-41	1092 ± 30	3.7 ± 0,2	1.0 ± 0.2	0.88 ± 0.02	0.94 ± n.a<br>0.09 ± 0.01

a) from N_2 nitrogen and BdB method, b) calculated from pore diameter and XRD assuming a hexagonal channel array, c) calculated at P/P_0 =0.90

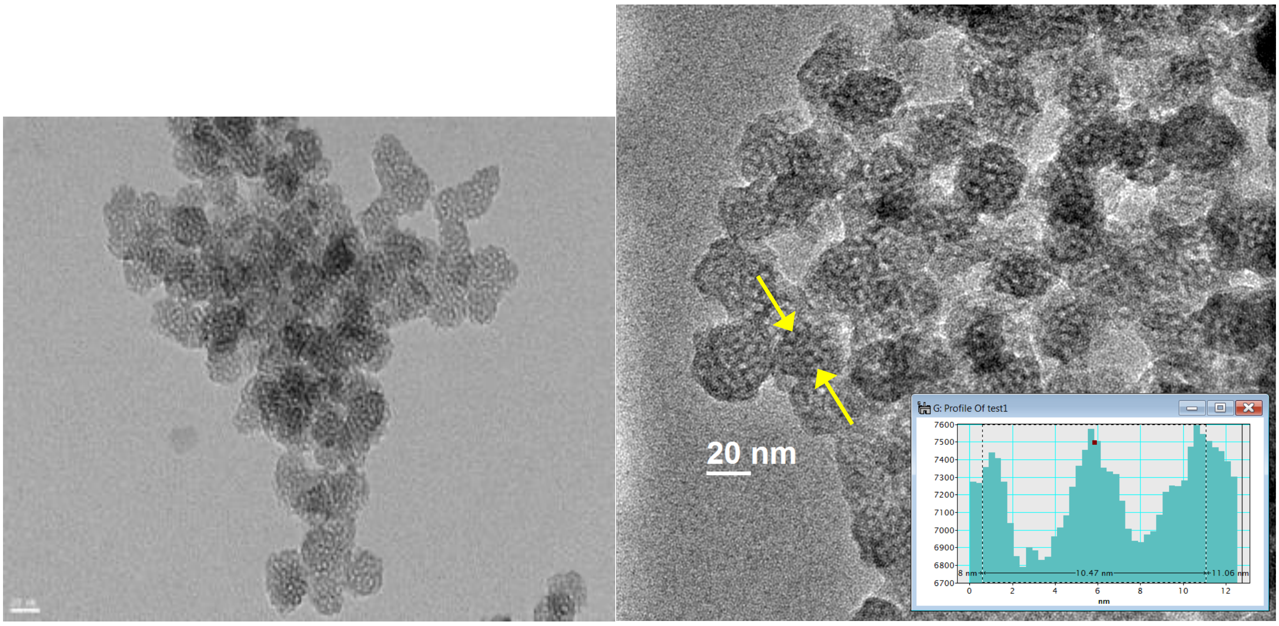

Figure. S1 HRT EM image of calcined : **MSN-23-A** typical (bottom) with insert for intensity profile analysis showing a distance 5.4 ± 0.2 nm between two pores and an average particle size diameter of 22 ± 3 nm prepared as calcined MSN-22-A (top image) with an average size of 22 nm ± 3 nm.

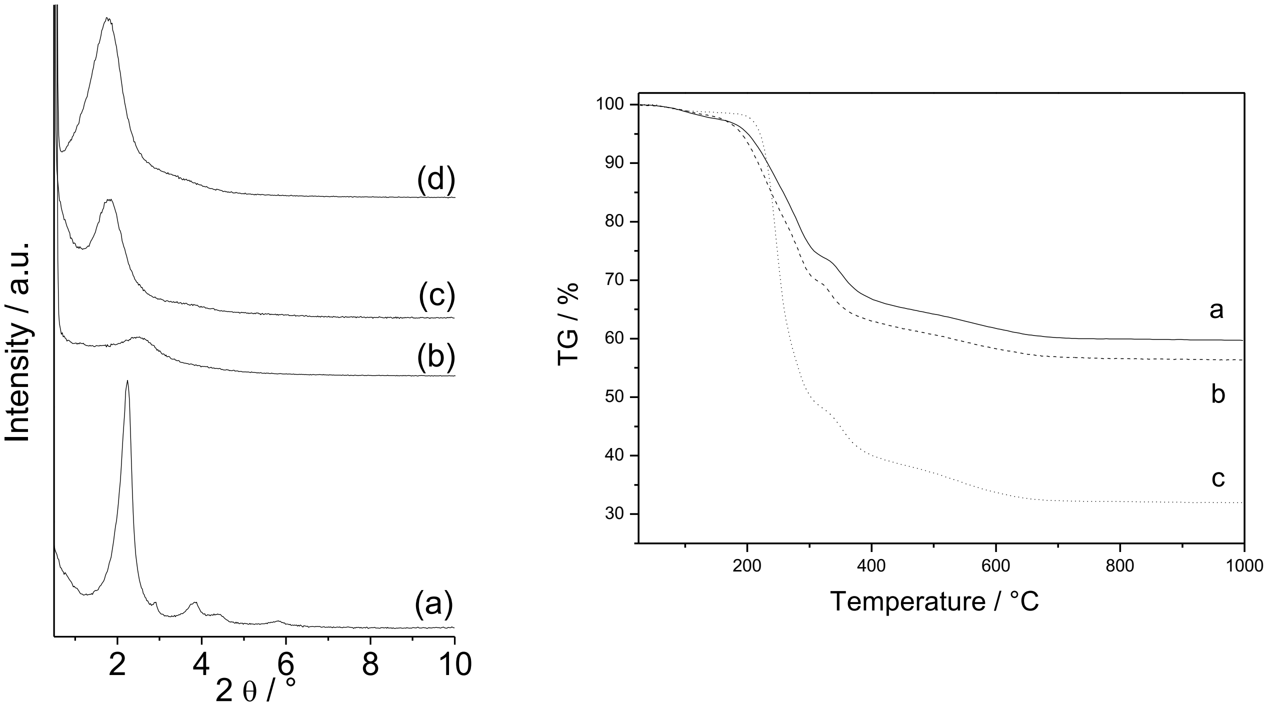

Figure. S2 (left) Comparison of XRD patterns of MSM-100 (a,b) and MSN-20 (c, d) as-synthesized (a, c) and calcined then treated in distilled water (b, d), i. e., i) calcined mesoporous silica powder 0.1 g + distilled water 10 g, stirred at RT overnight, ii) centrifugated and iii) dried at 50°C; (right) TGA of the as-synthesized nanoparticles of (a) **MSN-20-A**, (b) **MSN-100-A** acid treated (c) **MSN-100** (washed with deionized water).

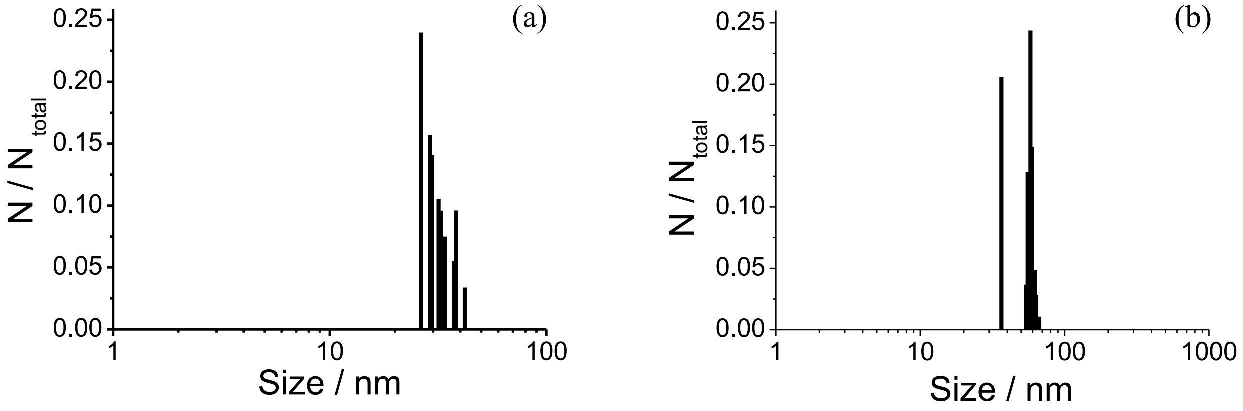

Figure. S3 DLS size distribution of **MSN-20-A**, measured after the synthesis with flocculate comprised between 24 and 41 nm (a) and 3 months later with a single peak at 37 and a distribution in the range 52-69 nm (b).

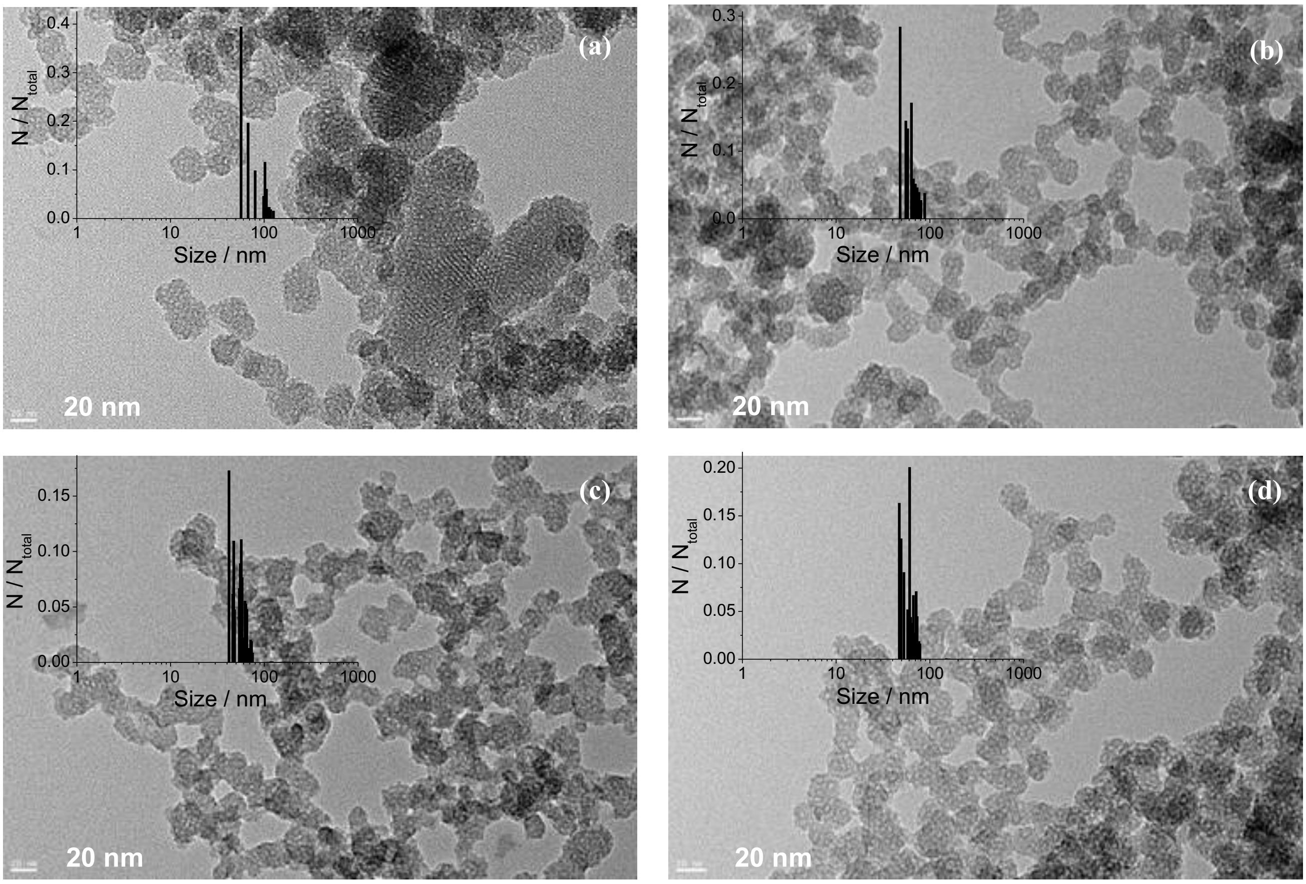

Figure. S4 TEM images of US-MSN obtained from the reaction solution at the molar ration of 1 SiO_2 : 0.5 NaOH : x CTAB : y F127 : 130 H_2O. (a) x = 0.12, y = 0.000104 (twice more F127), (b) x = 0.12, y = 0.000026 (twice less F127), (c) x = 0.24 (twice more CTA), y = 0.000052, and (d) x = 0.06 (twice

less CTA), y = 0.000052. Same nucleation quenching using dilution by 6 times. Inserts : corresponding DLS size distribution of the nanoparticle aggregates in the as-synthesized solution (a, b, c, and d).

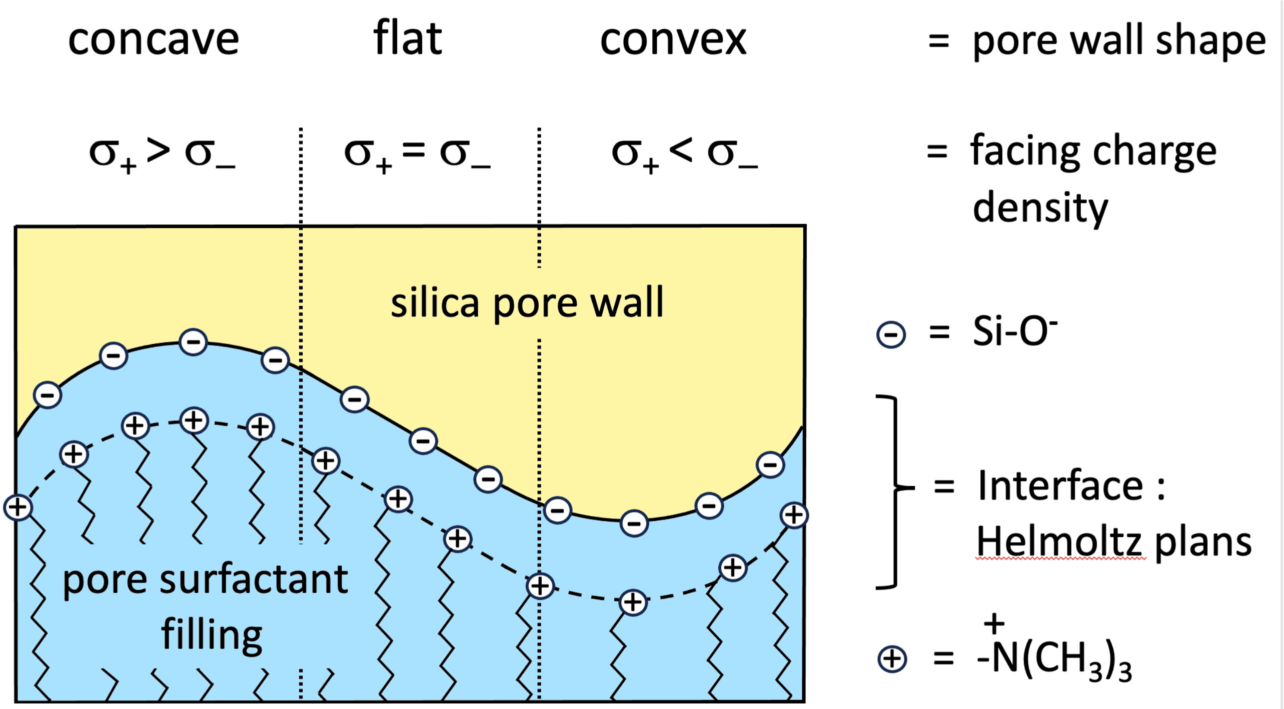

Figure. S5 Surface curvature generated by charge density mismatch between the Helmholtz plans in the silica-surfactant interface inspired from ref. 49 where σ_i and σ_s stand for charge densities on the silica pore wall (Si-O$^-$) and on the surfactant micelles palisade entrapped in the templated pores (positively charged ammonium heads).

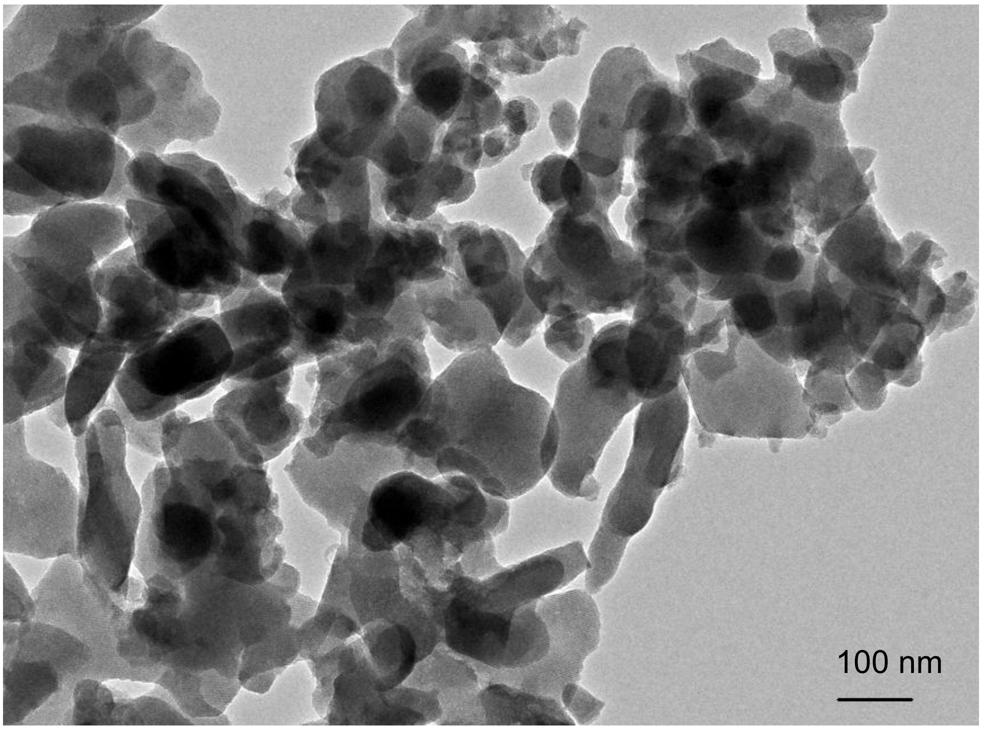

Figure. S6 TEM image of **MSN-100** synthesized in the presence of a large quantity of F127 as reported in ref.11 by Imai et al.; average particle size of 100 nm.
